# Human DNA Helicase B as a Candidate for Unwinding Secondary CGG Repeat Structures at the *Fragile X Mental Retardation* Gene

**DOI:** 10.3389/fnmol.2018.00138

**Published:** 2018-04-30

**Authors:** Gulfem D. Guler, Zev Rosenwaks, Jeannine Gerhardt

**Affiliations:** ^1^Celgene Quanticel Research, San Francisco, CA, United States; ^2^The Ronald O. Perelman and Claudia Cohen Center for Reproductive Medicine, Weill Cornell Medicine, Cornell University, New York, NY, United States; ^3^Department of Obstetrics and Gynecology, Weill Cornell Medicine, Cornell University, New York, NY, United States

**Keywords:** replication, helicases, fragile X syndrome, fragile sites, secondary structure, repeats

## Abstract

The fragile X syndrome (FXS) is caused by a CGG repeat expansion at the *fragile X mental retardation* (*FMR1*) gene. *FMR1* alleles with more than 200 CGG repeats bear chromosomal fragility when cells experience folate deficiency. CGG repeats were reported to be able to form secondary structures, such as hairpins, *in vitro*. When such secondary structures are formed, repeats can lead to replication fork stalling even in the absence of any additional perturbation. Indeed, it was recently shown that the replication forks stall at the endogenous *FMR1* locus in unaffected and FXS cells, suggesting the formation of secondary repeat structures at the *FMR1* gene *in vivo*. If not dealt with properly replication fork stalling can lead to polymerase slippage and repeat expansion as well as fragile site expression. Despite the presence of repeat structures at the* FMR1* locus, chromosomal fragility is only expressed under replicative stress suggesting the existence of potential molecular mechanisms that help the replication fork progress through these repeat regions. DNA helicases are known to aid replication forks progress through repetitive DNA sequences. Yet, the identity of the DNA helicase(s) responsible for unwinding the CGG repeats at *FMR1* locus is not known. We found that the human DNA helicase B (HDHB) may provide an answer for this question. We used chromatin-immunoprecipitation assay to study the *FMR1* region and common fragile sites (CFS), and asked whether HDHB localizes at replication forks stalled at repetitive regions even in unperturbed cells. HDHB was strongly enriched in S-phase at the repetitive DNA at CFS and *FMR1* gene but not in the flanking regions. Taken together, these results suggest that HDHB functions in preventing or repairing stalled replication forks that arise in repeat-rich regions even in unperturbed cells. Furthermore, we discuss the importance and potential role of HDHB and other helicases in the resolution of secondary CGG repeat structures.

## Introduction

The fragile X syndrome (FXS) is the most common inherited form of intellectual disability. FXS is caused by a CGG repeat expansion on the X chromosome in the 5’UTR of the *fragile X mental retardation (FMR1*) gene (Nelson et al., 2013). CGG repeat expansion leads to *FMR1* gene silencing. FXS is inherited from women carrying a premutation, 55–200 CGG repeats. More than 200 CGG repeats are categorized as a full mutation (FM), resulting in FXS (Kronquist et al., 2008). Furthermore, expansion of CGG repeats in premutation range within *FMR1* gene is also associated with other disorders; fragile X-associated primary ovarian insufficiency (FXPOI; Sherman, 2000), fragile X-associated diminished ovarian reserve (DOR; Man et al., 2017) and fragile X-associated tremor ataxia syndrome (FXTAS; Hagerman et al., 2001). In contrast to FXS patients, the *FMR1* gene is transcribed in premutation carriers. A toxic RNA gain-of-function and the expression of an abnormal FMRpolyglycin protein is suggested to cause the symptoms in premutation carriers (Hagerman and Hagerman, 2013; Todd et al., 2013).

The expanded repeats at the *FMR1* gene locus in FXS cells are characterized as rare fragile sites (RFS). While RFS are only found in some individuals, common fragile sites (CFS; Durkin and Glover, 2007) and early replicating fragile sites (ERFS; Barlow et al., 2013) are found in every individual. In contrast to RFS, CFS and ERFS contain non-expanding repetitive DNA sequences, and are therefore stable under normal conditions. However, RFS as well as CFS and ERFS tend to break upon replicative stress, which could result in chromosomal deletions, translocations and sister chromatid exchanges (Glover and Stein, 1987, 1988; Wang et al., 1997). It was suggested that these chromosomal alterations are consequences of prolonged replication fork stalling at repetitive DNA sequences located at these fragile sites.

It was reported that DNA and RNA containing CGG repeats are able to form secondary structures, such as hairpins (Gacy et al., 1995; Gacy and McMurray, 1998), G-quadruplexes (Fry and Loeb, 1994; Khateb et al., 2004) and R-loops (Groh et al., 2014; Loomis et al., 2014). DNA templates with such secondary structures stall replication forks *in vitro* and *in vivo* (Samadashwily et al., 1997; Pelletier et al., 2003; Voineagu et al., 2009; Gerhardt et al., 2014). Consistent with prolonged fork stalling at CGG repeats, replication of the *FMR1* locus is delayed in FXS cells compared to unaffected cells (Hansen et al., 1993; Subramanian et al., 1996). Prolonged replication fork stalling and uncompleted DNA replication at this fragile site can lead to genomic instability such as DNA break-induced chromosomal alterations or repeat expansions due to DNA polymerase slippage (Madireddy and Gerhardt, 2017). On the other hand, non-canonical secondary structures may turn CGG repeat containing FMR1 mRNA into toxic RNA, which may be pathogenic through sequestering RNA-binding proteins. Thus, molecular mechanisms that disrupt these secondary structures are crucial for genome stability and cellular function.

Here we discuss mechanisms that may prevent chromosomal fragility and repeat expansion at the* FMR1* locus upon replication fork stalling at CGG repeats, and the possible involvement of Human DNA helicase B (HDHB) at unwinding CGG repeat structures to aid replication fork progression. We present data that show HDHB localization to fragile sites specifically during S-phase even in unperturbed cells, suggesting that HDHB may help prevent or repair replication fork stalling. Furthermore, we discuss other possible DNA and RNA helicases that are capable of unwinding secondary CGG repeat structures.

## DNA Helicases Involved in Resolution of Secondary Repeat Structures During Replication Fork Progression

Despite the threats to genome stability posed by the secondary structures adopted by repeats, replication most often proceeds through repeat regions without the expression of chromosomal fragility. This suggests the presence of molecular mechanisms that help replication fork progression through repeat regions. At the heart of these mechanisms are DNA helicase(s) responsible for unwinding the secondary structures adopted by repeats. Helicases separate strands of a DNA double helix or a self-annealed RNA molecule using the energy from ATP hydrolysis, a process characterized by the breaking of hydrogen bonds between annealed nucleotide bases. DNA helicases translocate on double-stranded DNA (dsDNA) or single-stranded DNA (ssDNA) to unwind dsDNA into ssDNA or remove proteins bound to DNA. There is a plethora of helicases encoded by the genome, each performing a specialized function dictated by their enzymatic properties as well as their interaction partners.

**Figure 1:**
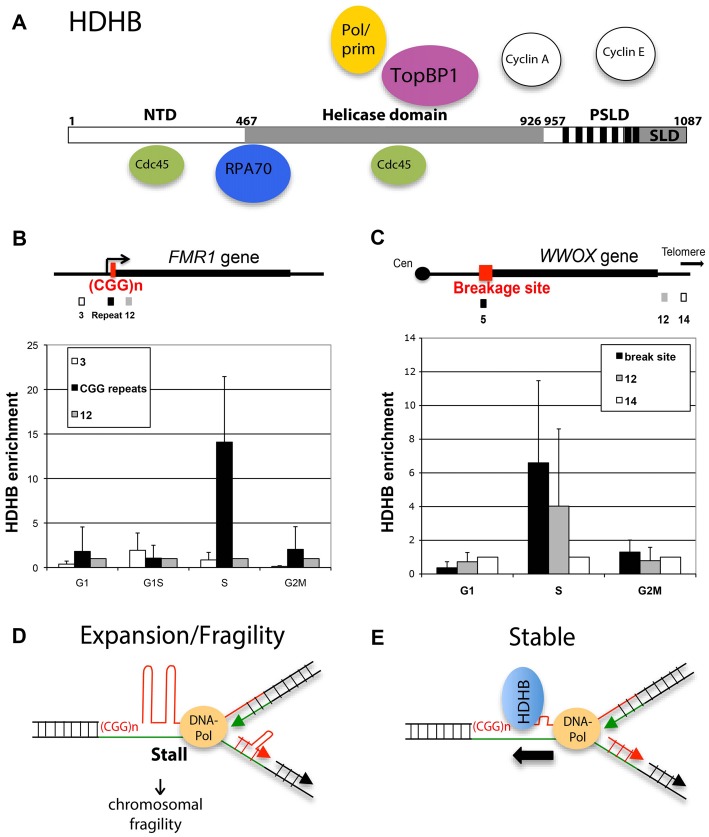
Human DNA helicase B (HDHB) resides at fragile sites in particular at the CGG repeats at human *fragile X mental retardation (FMR1)* gene locus. **(A)** Functional regions of HDHB. An N-terminal domain (NTD) is uncharacterized. The central domain (gray; residues 467–926) contains the seven superfamily I helicase motifs. The C-terminal domain contains consensus CDK phosphorylation sites (vertical black bars) and a subcellular localization domain (SLD), which together constitute a phosphorylation-dependent SLD (PSLD). Replication proteins, which are binding to HDHB are indicated. **(B)** Maps showing *FMR1* gene locus and three primer sets and **(C)** the common fragile sites (CFS) FRA16D and three primer sets. ChIP experiments were performed using U2OS cells synchronized in G1, S and G2/M cells and affinity-purified polyclonal HDHB antibody or rabbit IgG as control as described before (Gerhardt et al., 2006, 2015). HDHB enrichment at each site in each of three independent experiments is plotted on the Y-axis. Horizontal bars show the average HDHB enrichment at each time point and error bars are indicated. **(D,E)** Model for repeat expansion and fragility after fork stalling at secondary CGG repeat structures. HDHB could prevent genome instability by resolving the non-canonical repeat structures.

Conserved among vertebrates, DNA helicase B (DHB) contains seven helicase motifs of superfamily 1 with sequence similarity to homologous recombination proteins prokaryotic RecD and bacteriophage T4 dda. It works with 5’ to 3’ polarity. The C-terminus of HDHB contains a phosphorylation-dependent subcellular localization domain (PSLD; Figure 1A). PSLD is responsible for nuclear localization in G1-phase and phosphorylation-dependent nuclear export of HDHB at the G1/S transition (Gu et al., 2004). HDHB was shown to associate with pre-replication complex components Cdc45 and TopBP1 (Taneja et al., 2002; Gerhardt et al., 2015). HDHB also interacts with Cyclin E and A (Gu et al., 2004) and ssDNA-binding protein RPA (Guler et al., 2012). It was reported that its helicase activity is necessary for replication initiation (Taneja et al., 2002). At G1/S, majority of HDHB is exported from the nucleus. However, a low level of HDHB is retained on bulk chromatin during S-phase and this fraction is increased in cells exposed to agents that stall fork progression. Furthermore, DNA damage leads to HDHB accumulation on chromatin particularly during S-phase (Guler et al., 2012). Single stranded DNA-bound RPA at stalled replication forks recruit HDHB (Figure 1A) by a direct protein interaction between HDHB and RPA.

In addition to HDHB, there may be other helicases to help manage replication stress induced by repeat structures. Particularly, helicases capable of resolving G-quadruplexes adopted by guanine-rich stretches of DNA such as CGG repeats are of interest. G-quadruplexes may require specialized machinery to unwind them so that replication fork can progress through. One of the helicases capable of resolving G-quadruplexes is another superfamily 1 member with sequence similarity to RecD, called Pif1 helicase. Closely related to *S. cerevisiae* Rrm3 helicase, Pif1 was shown to promote replication fork progression through genomic regions that contain G-quadruplex sites (Paeschke et al., 2011). Furthermore, Pif1 functions to prevent G-quadruplex-associated DNA damage (Paeschke et al., 2013). Another helicase implicated at resolving G-quadruplexes is Dna2 nuclease-helicase, which also has roles in telomere maintenance in addition to Okazaki fragments-processing while traveling with the fork (Lin et al., 2013). Moreover, RecQ helicase members Bloom’s syndrome helicase (BLM), Werner’s syndrome helicase (WRN), and Fanconi anemia group J (FANCJ) helicase are all capable of resolving G-quadruplexes and accumulate at stalled replication forks (London et al., 2008). Of particular note, WRN was previously reported to unwind CGG repeats *in vitro* (Fry and Loeb, 1999). Even though none of these helicases were yet shown to associate with the *FMR1* gene, it remains to be investigated whether these helicases can help replication forks progress through the CGG repeats at the *FMR1* locus.

## HDHB Is Recruited to the Repeats at the *FMR1* Gene and at Common Fragile Sites

Replication forks may stall naturally without any additional perturbation at hard to replicate regions such as repetitive regions. Replication fork stalling initially results in long stretches of ssDNA that get coated with RPA. To facilitate replication fork recovery, ssDNA-bound RPA recruits DNA damage response proteins, including helicases like HDHB (Guler et al., 2012).

To investigate whether HDHB associates with replication forks stalled at repetitive DNA sequences, in particular to the CGG repeats at *FMR1* locus and AT-rich repeats at FRA16D, we performed chromatin immunoprecipitation (ChIP) experiments. U2OS cells were blocked in G2/M-phase and released for 5 h (G1), or synchronized in G1/S-phase and released for 6 (S) or 9 h (G2/M), followed by formaldehyde treatment to crosslink chromatin. Chromatin was isolated, sheared and immunoprecipitated using purified polyclonal HDHB antibody or non-immune control antibody (Gerhardt et al., 2015). HDHB enrichment in immunoprecipitated chromatin was measured by quantitative real-time PCR using one primer pair amplifying DNA segment containing the repeat region/break site (Figures 1B,C) and two primer pairs in a distal region (*FMR1* gene: primer pair 3 and 12 (Gray et al., 2007); FRA16D/*WWOX* gene primer pair 12 and 14). In S-phase cells, HDHB was significantly enriched on chromatin in the repeat region of fragile sites relative to the distal regions. HDHB enrichment in both repeat regions was reduced in G1- and G2/M-phase. We found similar results at a second CFS, FRA3B (data not shown). These results show that HDHB is enriched in S-phase at the repetitive DNA sequences and suggest that HDHB is recruited to stalled replication forks at repetitive regions such as CGG repeats at the *FMR1* gene.

Upon recruitment to the fork, HDHB can unwind the secondary repeat structures formed by CGG repeats ahead of DNA-polymerase (Figures 1D,E). Repeat expansion following DNA polymerase slippage at stalled replication forks can be so prevented (Figure 1E) as well as chromosomal fragility, which could result from DNA breaks induced by replication fork stalling. It would be interesting to determine whether FXS and premutation patients have a decreased HDHB protein level or HDHB helicase activity, and whether such differences can affect replication fork stalling, chromosomal fragility and CGG repeat expansion.

## RNA Helicases Involved in Prevention of Secondary CGG Repeat RNA Structures

The presence of FMR1 mRNA in intranuclear inclusions (Tassone et al., 2004) in premutation patients, as observed in brain tissues from FXTAS patients (Galloway and Nelson, 2009), and increased FMR1 mRNA level in PM carriers (Tassone et al., 2000) led to the suggestion that a toxic RNA gain-of-function mechanism might be responsible for FXTAS development. An increased FMR1 mRNA level was also noticed in ovarian granulosa cells of female carriers with a PM (Elizur et al., 2014). Since RNAs containing CGG repeats can adopt secondary structures *in vitro* (Napierala et al., 2005), it is likely that FMR1 mRNA assumes non-canonical RNA structures *in vivo* as well.

It was previously shown that the expansion to FM is reduced by AGG interruptions within the premutated allele (Yrigollen et al., 2012; Nolin et al., 2014). AGG interruptions are located at the 5’ end of the CGG repeat sequence in *FMR1* gene (Kunst and Warren, 1994; Kunst et al., 1996, 1997). These interruptions were proposed to stabilize the repeats (Nelson et al., 2013), potentially by preventing the formation of secondary repeat DNA structures within the cell. AGG interruptions could also prevent formation of secondary structures in FMR1 mRNA. Indeed, we recently found that an increase in number of AGG interruptions, from none or one to two, is associated with lower risk of FXDOR in patients carrying a PM (Lekovich et al., 2017). We propose a model where AGG interruptions lowers the probability of secondary repeat structure formation in FMR1 mRNA, and hence pathogenesis, in the ovaries of women carrying a PM.

Other than cis-acting elements like AGG interruptions described above, RNA helicases could also prevent repeat-containing RNA to form non-canonical secondary structures. One candidate for unwinding rCGG repeat structures is Rm62, *Drosophila* ortholog of p68/DDX5 RNA helicase. p68 RNA helicase is a prototypical DEAD-box RNA helicase that has been implicated in transcriptional regulation, pre-mRNA splicing, and nucleocytoplasmic shuttling. Rm62 overexpression rescued neurodegeneration in flies expressing 90 CGG repeats (Qurashi et al., 2011). Additionally, RNA helicases p68/DDX5 and DDX6 were reported to unwind expanded CUG repeats in myotonic dystrophy (Laurent et al., 2012; Pettersson et al., 2014), making these helicases of therapeutic interest. Another helicase able to unwind G-quadruplexes and R-loop structures is RNA helicase A (RHA, also called DHX9, and nuclear DNA helicase II). RHA unwinds DNA–DNA, RNA–RNA and DNA–RNA duplexes with 3’ to 5’ direction. It acts preferentially on RNA substrates.

Additional proteins, involved in preventing the formation of secondary RNA structures, are heterogeneous nuclear ribonucleoproteins A2/B1 (hnRNP A2/B1). HnRNP A2/B1 have a role in packaging nascent mRNA, alternative splicing and cytoplasmic RNA trafficking, translation and stabilization. Found in intranuclear inclusions of FXTAS patients (Iwahashi et al., 2006), hnRNP A2/B1 were described to act as an RNA chaperone destabilizing RNA structures formed by CGG repeats (Khateb et al., 2004; Ofer et al., 2009). HnRNP A2/B1 overexpression rescues neurodegeneration in *drosophila* expressing 90 CGG repeats (Jin et al., 2007; Sofola et al., 2007). It remains to be determined if these helicases or proteins unwinding secondary structures are deregulated in patients and whether helicase deficiency promotes the pathogenicity observed in patients with a premutation.

## Conclusion

Repeats present a challenge for the replication machinery and other cellular processes involving repetitive DNA and RNA. Therefore, mechanisms to resolve secondary structures that repeats might adopt are needed. Specialized helicases are able to unwind secondary DNA and RNA structures, which can otherwise lead to replication fork stalling or toxic RNA, respectively. Replication fork stalling at endogenous repeats is reported in cells derived from FXS and FRDA patients (Gerhardt et al., 2014, 2016). This can lead to genomic instability by DNA polymerase slippage-induced repeat expansion or chromosomal fragility when occurred at RFS and CFS (Madireddy et al., 2016). Toxic RNAs, on the other hand, may disrupt cellular processes particularly by sequestering proteins important for RNA function. Therefore, resolution of secondary structures, which DNA or RNA repeats adopt, by DNA/RNA helicases is a crucial mechanism that could help prevent repeat-induced diseases. Consequently, helicases that resolve such secondary structures adopted by DNA or RNA repeats may constitute a crucial toolbox cells employ to help prevent repeat-induced diseases such as fragile X syndrome.

## Author Contributions

GG and JG performed the experiments. GG, JG and ZR wrote and edited the manuscript.

## Conflict of Interest Statement

GG is an employee of Celgene. The other authors declare that the research was conducted in the absence of any commercial or financial relationships that could be construed as a potential conflict of interest.
